# Assessment of the toll-like receptor 4 Asp299Gly, Thr399Ile and interleukin-8 -251 polymorphisms in the risk for the development of distal gastric cancer

**DOI:** 10.1186/1471-2407-7-70

**Published:** 2007-04-26

**Authors:** Elvira Garza-Gonzalez, Francisco J Bosques-Padilla, Sandra I Mendoza-Ibarra, Juan P Flores-Gutierrez, Hector J Maldonado-Garza, Guillermo I Perez-Perez

**Affiliations:** 1 Departamento de Microbiología, Facultad de Medicina, Universidad Autónoma de Nuevo León, Monterrey, Mexico; 2 Servicio de Gastroenterología, Hospital Universitario "Dr. José Eleuterio González", Universidad Autónoma de Nuevo León, Monterrey, Mexico; 3 Laboratorio de Anatomía Patológica, Hospital Universitario "Dr. José Eleuterio González" Universidad Autónoma de Nuevo León, Monterrey, Mexico; 4 Departments of Medicine and Microbiology, New York University School of Medicine, New York, USA

## Abstract

**Background:**

The intensity of the inflammation induced by *Helicobacter pylori *colonization is associated with the development of distal gastric cancer (GC). The host response to *H*. *pylori *has been related to genetic polymorphisms that influence both innate and adaptive immune responses.

Our aim was to investigate whether the presence of the *TLR4 Asp299Gly*, *TLR4 Thr399Ile *and *IL-8-251 *A/T polymorphisms had any influence in the development of distal GC in a Mexican population.

**Methods:**

We studied 337 patients that were divided in two groups: 78 patients with histologically confirmed distal GC and 259 non-cancer controls. The presence of *H. pylori *in the control population was defined by positive results of at least two of four diagnostic tests: serology, histology, rapid urease test and culture. Human DNA was purified and genotyped for *TLR4 Asp299Gly *polymorphism by pyrosequencing, for *TLR4 Thr399Ile *by PCR-RFLP and for *IL8-251 *by the amplification refractory mutation system (ARMS)-PCR.

**Results:**

The non-cancer control group was found to be in Hardy-Weinberg equilibrium at the polymorphic loci studied (chi-square _H-W _= 0.58 for *IL8-251*, 0.42 for *TLR4 Asp299Gly *and 0.17 for *TLR4 Thr399Ile*). The frequencies of mutated alleles (homozygous plus heterozygous) were compared between cases and controls. We found no significant difference for *TLR4- Asp299Gly *[the 7.7% of distal GC patients and 7.7 % non-cancer controls (p = 0.82)] and for *TLR4 Thr399Ile *[the 1.3% of GC patients and the 5% of the control population (p = 0.2)]. In contrast, for *IL-8-251 *A/T, 80.77% of the GC patients and 66.4% in the control group age and gender matched had at least one copy of mutated allele (OR = 2.12, 95% CI = 1.1–4.2) (p = 0.023).

**Conclusion:**

This study showed that the IL8-251*A allele could be related to the development of distal gastric cancer in this Mexican population.

## Background

*Helicobacter pylori *almost exclusively colonize the mucous layer of the human stomach and reside in the host for years, decades or maybe lifelong. In approximately 10% of cases, the organism is associated with diverse clinical outcomes, including non-ulcer dyspepsia, peptic ulcer disease, and distal gastric cancer [1].

Both host and environmental factors have been associated with this clinical diversity. Host factors are mainly related to the recognition of the bacteria by the immune system and variation in the level of cytokine response [2,3].

Recognition of pathogens is mediated by a set of germline-encoded receptors that are referred to as pattern-recognition receptors (PRRs). These receptors recognize conserved molecular patterns, which are shared by large groups of microorganisms. An important PRR is Toll-like receptor 4 (TLR4), a transmembrane receptor that recognizes a range of ligands, including lipopolysaccharide (LPS), which is found in the cell wall of Gram-negative bacteria like *H. pylori *[3]. Two single nucleotide polymorphisms (SNPs) in the *TLR4 *gene, Asp-299→Gly and Thr-399→Ile transitions, have been shown to lead to hyporesponsiveness to LPS, reduced epithelial TLR4 density and reduced inflammatory cytokine response to LPS [4]. Furthermore, genetic variants of cytokines are also critical for the inflammatory response, and several genes from different pathways have been associated with gastric cancer, including interleukin *(IL)-1B*, *TNF*, *IL-10 *and *IL-8 *[5-7].

IL-8 cytokine functions as a potent chemoattractant for neutrophils and lymphocytes. It has been reported that IL-8 levels increased 10-fold in gastric cancer specimens when compared with normal gastric tissues. A well-characterized SNP T/A in the -251 locus has been described and associated with higher IL-8 production and several diseases [8-11].

Our aim was to determine whether the *TLR4 Asp299Gly*, *TLR4 Thr399Ile *and the *IL8-251 A/T *polymorphisms are associated with distal gastric cancer in a well-defined Mexican population.

## Methods

### Patient population

We studied 78 unrelated patients with histologically confirmed distal GC (mean age = 58.6y, median age = 60, age range: 22–84, F:M = 0.56). We also studied 259 patients with no histological evidence of GC (mean age = 57.1y, median age = 54, age range = 18–92, F:M = 1.17). This population has been described previously [7]. All patients were enrolled at the Hospital Universitario "Dr. José Eleuterio González", Universidad Autónoma de Nuevo León. The local ethical committee approved the study and written informed consent was obtained from all subjects.

### Histopathological examinations

From all control patients, eight biopsy specimens were obtained for histological evaluation, two from the lesser curvature, two from the greater curvature, two from the incisura angularis, and two from the prepyloric region. From patients with distal GC, at least 8 biopsies were obtained from the tumor for histological evaluation. Biopsies from cases and controls were fixed, paraffin embedded and stained with hematoxilin-eosin. An experienced pathologist examined all the histological slides.

### *H. pylori *status

The *H. pylori *status of the control population was determined by histology, the rapid urease test (RUT), serology and culture. For the RUT, an antral biopsy was analyzed by a validated non-commercial test [12]. The biopsy was placed in a test vial, incubated at 37°C and read within 24 h. A color change from orange to magenta indicated a positive result. For serology, an enzyme-linked immunosorbent assay (ELISA) was used to demonstrate the presence of IgG antibodies as previously described [13].

Culture was performed on two corpus and two antrum biopsies by standard methods. Biopsies were placed in 10% blood agar (Becton Dickinson, Cockeysville, MD) and incubated at 37°C for at least one week under microaerobic conditions. Strains were identified by Gram staining, oxidase, catalase and urease tests.

Control patients were considered *H. pylori *infected when at least two of the diagnostic tests were positive.

The prevalence of *H. pylori *infection in gastric cancer patients was determined by the result of histology only.

### Genotyping

Genomic DNA was extracted from peripheral blood by the phenol-chloroform-isoamilic alcohol and precipitation with ethanol method. An amplification refractory mutation system (ARMS)-PCR method was used to genotype the *IL8-251 *polymorphism as described previously [14].

The *TLR4 Thr399Ile *polymorphism was determined by PCR-RFLP [15] and the *TLR4 Asp299Gly *polymorphism was determined by a pyrosequencing method designed for this study. Initially, a PCR was performed with the primers F-GAT TAG CAT ACT TAG ACT ACT ACC TCC ATG and R-CCC TTT CAA TAG TCA CAC TCA CCA GG with the reverse primer biotinylated. The PCR conditions were: 94°C for 5 min and 40 cycles of 94°C, 59°C and 72°C for 30 s each, and a final extension at 72°C for 5 min. For pyrosequencing, biotinylated PCR templates were immobilized on streptavidin-coated paramagnetic Sepahrose beads in binding buffer. The bead-template complexes were washed by submersion in 70% alcohol and 0.5 M NaOH and then added to 45 μl of annealing buffer containing 15 pmol of the sequencing primer 5'-CAT ACT TAG ACT ACT ACC TC 3'. Annealing took place at 80°C for 2 min. Real-time pyrosequencing was performed in an automated 96-well pyrosequencer PSQ SNP 96 (Pyrosequencing AB, Uppsala, Sweden) using enzymes and substrates recommended by the manufacturer.

### Statistical analysis

Hardy-Weinberg equilibrium of alleles at individual loci was assessed by chi-square statistics. Statistically significant differences were determined by Student's *t *test, chi-square or Fisher exact test, two tailed. A probability (p) value < 0.05 was considered as statistically significant. Odds ratios (OR) with 95% confidence intervals (CIs) were computed using the Epi-Info 2000 software (Center for Disease Control and Prevention, Atlanta, Ga.). Three analyses were performed, one in which all cases and controls were included, a second in which cases were compared only with *H. pylori *+ controls and a third analysis in which the population-based controls were matched by 5-year age group and gender with the cancer cases.

## Results

### Study groups characteristics and *H. pylori *status

The distribution of genotypes according to histological findings is shown on Table 1.

The genotype frequencies at the individual loci studied were in Hardy-Weinberg equilibrium, with non-significant chi-square values (0.58 for *IL8-251*, 0.42 for *TLR4 Asp299Gly *and 0.17 for the *TLR4 Thr399Ile*). Among the control group, the 80% had chronic gastritis (Table 1), 11.2% of patients had PUD and 8.8% of patients had atrophic gastritis and/or intestinal metaplasia. The proportion of diffuse and intestinal gastric cancer was 1.2:1.

The prevalence of *H. pylori *in the control group was 73.7%. This is similar to the value previously reported for this study population [16]. The prevalence of *H. pylori *among the gastric cancer patients was 53.8%.

### Assessment of risk of *IL8-251 *A/T, *TLR4 Asp299Gly *and *TLR4 Thr399Ile *genotypes

We compared all particular genotypes for *IL8-251 *(AA, AT and TT), for *TLR4 Asp299Gly *(1,1, 1,2 and 2,2) and *TLR4 Thr399Ile *(1,1, 1,2 and 2,2) (Table 1). The frequency of the *IL8-251 *AT genotype was higher in the gastric cancer cases than in all controls, only *H. pylori *positive controls and age and gender matched controls (Table 2).

### Assessment of risk of IL8-251*A, TLR4 Asp299Gly*2 and TLR4 Thr399Ile*2 alleles

We next compared the carriage of the IL8-251*A allele (homozygous plus heterozygous) for GC cases and all gastritis plus normal controls. We found an association of the IL8-251 *A allele and the development of GC (OR = 2.12, 95% CI = 1.1–4.2, p = 0.023) (Table 2). When we compared all gastritis plus normal controls *vs *histological cancer types, the risks for diffuse and intestinal gastric cancer types were similar but not significant (OR = 2.15, p = 0.08 and OR = 2.1, p = 0.13 respectively).

When we compared the gastric cancer cases with the *H. pylori*+ controls we found a similar value (OR = 2.12, 95% CI = 1.1–4.2, p = 0.029).

Next, we compared the frequency in the gastric cancer group withage and gender matched controls and we found a stronger association (OR: 2.42, 95% CI = 1.2–4.8, p = 0.0.009).

We did not find any association for TLR4 Asp299Gly*2 and TLR4 Thr399Ile*2 hyporeactive alleles.

## Discussions and conclusion

The diversity in response to the infection by *H. pylori *has been attributed to several factors. The genetically regulated immune response seems to play a crucial role in determining the intensity of damage to the host [1-3].

Several authors have described the increased production of IL-8 during *H. pylori *infection. An A/T polymorphism at the position -251 in the gene has been associated to a higher activity of this interleukin and recently, this allele has been related in two studies to the development of gastric cancer in Japanese population [10,11]. In one of those studies, the *IL8-251 AA *genotype held a higher risk of atrophic gastritis (OR = 2.35; 95%, CI = 1.12–4.94) and gastric cancer (OR = 2.22; 95% CI = 1.08–4.56) compared with the TT genotype. They studied 252 healthy controls, 215 individuals with atrophic gastritis, and 396 patients with gastric cancer. The AA and AT genotypes were significantly associated with higher levels of IL8 protein, more severe neutrophil infiltration compared with the TT genotype. In the other study, the IL-8 -251*A allele was associated to a higher risk of gastric cancer (OR = 2.1, 95% CI = 1.38–2.92). Our results showed no association between higher rates of GC and the AA genotype, but a positive association was found for the AT genotype and the carriage of at least one copy of the *A allele (homozygous plus heterozygous). It is remarkable that the ORs value observed in our study (2.12) was quite similar to that observed in the Japanese population (2.1).

The *IL-1B-31 C/T *polymorphism has been associated with the development of gastric cancer and a geographical-ethnic difference has been described for this locus, with a stronger association of the polymorphism in occidental than in oriental population. The results for the *IL-8-251 *A/T polymorphism correlate with previous data presented for Japanese population, showing no trend in geographical or ethnic variation.

It is currently unresolved whether a hyporesponsive LPS signaling pathway is beneficial or detrimental to the host. Individuals with the hyporeactive *TLR4 *polymorphisms have shown reduced levels of circulating inflammatory cytokines, an increased risk of acute bacterial infections and a trend towards increased mortality. Other recent studies support the hypothesis that the low-functioning *TLR4 *polymorphisms may result in a reduced inflammatory response associated with a low-damaging infection that will promote a persistent infection [17-20]. In this study, we did not find any association between GC and TLR4 polymorphisms, suggesting they do not contribute to the development of distal gastric cancer. Both *TLR4 Asp299Gly *and *Thr399Ile *polymorphisms have been reported in linkage disequilibrium (LD) [21,22], but we decided to genotype both because we did not know if they were in LD in our population.

In conclusion, our preliminary results suggest that *IL8-251 *is a biologically plausible disease-modifying polymorphism. If confirmed in multiple populations, as it has been in Japanese, and now Mexican groups, this polymorphic site, in conjunction with others, could be used to identify patients at greater risk for GC, who may need closer monitoring.

## Competing interests

The author(s) declare that they have no competing interests.

## Authors' contributions

EGG designed and coordinated the study, optimized PCR conditions, carried out molecular analysis and drafted the manuscript. SM performed PCRs analysis, JPFG performed histological examination and interpretation of results, FJBP and HJMG recruited patients and carried out endoscopies and gastric biopsies, GIPP conceived of the study, and participated in its design and coordination. All authors read and approved the final manuscript.

## Pre-publication history

The pre-publication history for this paper can be accessed here:



## Figures and Tables

**Table 1:** Genotype and allele frequencies of the *IL-8 251 *A/T and *TLR-299 *and *-399 *loci in the non-cancer and cancer groups according to histological findings

	*IL8-251 A/T*					*TLR4 Asp299Gly*				*TLR4Thr399Ile*			
	
Group (n)	genotype			allele		genotype		allele		genotype		allele	
	
	AA	AT	TT	*A	*T	1,1	1,2	*1	*2	1,1	1,2	*1	*2
Gastritis (207)	42	97	68	0.44	0.56	190	17	0.96	0.04	196	11	0.97	0.03
PUD (29)	4	14	11	0.38	0.62	29	0	1	0	27	2	0.97	0.03
AG + IM (23)	5	10	8	0.43	0.57	20	3	0.93	0.07	23	0	1	0
Gastric cancer (78)	16	47	15	0.51	0.49	72	6	0.96	0.04	77	1	0.99	0.01
diffuse type	8	26	8	0.5	0.5	38	4	0.95	0.05	41	1	0.99	0.01
intestinal type	8	21	7	0.51	0.49	34	2	0.97	0.03	36	0	1	0
*H. pylori *+ (191)	37	90	64	0.43	0.57	175	16	0.96	0.04	181	10	0.97	0.03
Age and gender matched (n = 189)	33	87	69	0.4	0.6	175	14	0.96	0.04	179	10	0.97	0.03

PUD: peptic ulcer disease; AG, atrophic gastritis; IM: intestinal metaplasia

**Table 2:** Odds ratios and 95% confidence intervals for the analysis the genotypes of distal gastric cancer cases *vs *all non-cancer controls.

	IL8-251*A		*IL8-251 AT*		TLR-299*2		TLR-399*2	
All cases *vs*	OR (95% CI)	p	OR (95% CI)	p	OR 95% CI	p	OR 95% CI	p

All controls (n = 259)	2.12 (1.1–4.2)	0.023	1.73 (1–3)	0.049	1 (0.3–2.8)	.82	0.25 (0.01–1.8)	0.2
*H. pylori *+ controls (n = 191)	2.12 (1.1–4.2)	0.029	1.7 (0.96–3)	0.068	0.91 (0.3–2.6)	0.95	0.24 (0–1.8)	0.19
Age and gender matched controls (n = 189)	2.42 (1.2–4.8)	0.009	1.78 (1–3.2	0.035	1.04 (0.3–3.1)	0.86	0.23 (0–1.8)	0.1
